# Changes in ascending aorta dimensions, aortic valve function and systolic ventricular function over time in patients with congenital aortic stenosis

**DOI:** 10.1186/1532-429X-13-S1-P211

**Published:** 2011-02-02

**Authors:** Alexia Rossi, Denise van der Linde, Tirza Springeling, Adriaan Moelker, Gabriel P Krestin, Robert J van Geuns, Jolien Roos-Hesselink

**Affiliations:** 1 Erasmus MC, Rotterdam, Netherlands

## Introduction

Bicuspid aortic valve (BAV) is one of the most common congenital heart malformations and is a frequent cause of aortic valve stenosis (AoS).

## Purpose

The aim of this study was to evaluate the progression of aortic dilatation, aortic valve function, and ventricular function in patients with congenital AoS.

## Methods

Twenty-five patients with congenital AoS were prospectively followed with ECG-gated cine-MRI. Aortic root and ascending aorta diameters were measured. Peak aortic velocity and mean gradient were assessed using a retrospectively gated phase contrast sequence. Left ventricular hemodynamic parameters and myocardial mass were calculated using steady-state free precession images.

## Results

Sixteen men and 9 women were included in the study (mean age 32.6±7.6 years). The mean time (±SD) between baseline and follow-up study was 33.7±6.1 months. During follow-up aortic diameters significantly increased at aortic annulus by 1.4 mm (p=0.014), at sinus of Valsava by 2.0 mm (p=0.003) and at ascending aorta level by 2.0 mm (p<0.001). No significant increase in sinotubular-junction diameter was observed. Peak velocity significantly increased from 329.1±70.2cm/s at baseline to 353.4±82.6cm/s at follow-up with a mean progression of 8.0 cm/s/year. Mean aortic valve gradient increased significantly from baseline to follow-up: 44.5±18.6 vs 53.5±25.3 mmHg (p=0.021). Ejection fraction significantly increased from 57.6±6.4 to 59.7±7.1% (p=0.001) and a significant increase of myocardial ventricular mass was observed from 129.2±36.2 at baseline to 132.9±37.8 grams at follow-up (p=0.015).

## Conclusions

After a mean follow-up of 33 months a progression of aortic diameter dilation, aortic peak velocity, mean gradient and ventricular mass occurred in adult patients with congenital AoS.

